# Being bound and tied by the ropes of frugality: a case study on public management values and service integration

**DOI:** 10.1108/JHOM-10-2020-0401

**Published:** 2022-03-16

**Authors:** Therese Dwyer Løken, Marit Kristine Helgesen, Halvard Vike, Catharina Bjørkquist

**Affiliations:** Faculty of Health, Welfare and Organisation, Østfold University College , Halden, Norway; Department of Health, Social and Welfare Studies, Faculty of Health and Social Sciences, University of South-Eastern Norway , Kongsberg, Norway

**Keywords:** Service integration, Public management values, Organizational structures, Financial structures, Professional practice, Mental health and substance abuse

## Abstract

**Purpose:**

New Public Management (NPM) has increased fragmentation in municipal health and social care organizations. In response, post-NPM reforms aim to enhance integration through service integration. Integration of municipal services is important for people with complex health and social challenges, such as concurrent substance abuse and mental health problems. This article explores the conditions for service integration in municipal health and social services by studying how public management values influence organizational and financial structures and professional practices.

**Design/methodology/approach:**

This is a case study with three Norwegian municipalities as case organizations. The study draws on observations of interprofessional and interagency meetings and in-depth interviews with professionals and managers. The empirical field is municipal services for people with concurrent substance abuse and mental health challenges. The data were analyzed both inductively and deductively.

**Findings:**

The study reveals that opportunities to assess, allocate and deliver integrated services were limited due to organizational and financial structures as the most important aim was to meet the financial goals. The authors also find that economic and frugal values in NPM doctrines impede service integration. Municipalities with integrative values in organizational and financial structures and in professional approaches have greater opportunities to succeed in integrating services.

**Originality/value:**

Applying a public management value perspective, this study finds that the values on which organizational and financial structures and professional practices are based are decisive in enabling and constraining service integration.

## Introduction

Health and social care reforms are aimed at organizational renewal ( Pollitt, 2013 ), and they carry public management values that change both working conditions for professionals and services for service recipients ( Franco *et al.* , 2002 ). The purpose of this study is to demonstrate how these values influence service integration in municipal health and social care organizations.

As in other Western countries, several elements in the health and social care reforms in Norway have been inspired by values of New Public Management (NPM) ( Nesvaag and Lie, 2010 ). These reforms affected organizational and financial structures by dividing public health and social care organizations into single- or few-purpose organizations with separate funding streams, each pursuing defined sets of goals and tasks ( Christensen and Lægreid, 2007 ). The reforms also increased municipal responsibilities, placing professionals and managers under great pressure to achieve tight financial discipline and to organize their services in a cost-effective manner ( Vabø, 2009 ). In response, later health and social care reforms known as post-NPM aimed to address the challenges brought on by fragmentation, by enhancing organizational integration and collaboration ( Læ *greid et al.* , 2015 *)* . Although these reforms offer additional governance concepts to guide the complex issues that public sector organizations are required to handle, fragmentation due to organizational structures still is a challenge ( Steihaug *et al.* , 2016 *).*

Integrated health and social care services have been promoted as a means to improve access, quality and continuity of services in a more efficient way ( Valentijn *et al.* , 2013 ). This can be done through a coherent set of methods and models on the funding, administrative, organizational, service delivery and clinical levels in order to create connectivity, alignment and collaboration within and between organizational units ( Kodner and Spreeuwenberg, 2002 ). This implies delivery of coordinated services that require collaboration, along with effective and efficient information transfer and resource management. This enables professionals to ensure that services are holistic, person-centered, relevant, well planned and supportive of self-management *(* Ehrlich *et al.* , 2009 ; Montenegro *et al.* , 2011 ).

Without integration, service recipients risk poor access to services, lack of continuity and inadequate provision for their needs ( Montenegro *et al.* , 2011 ). Integration is especially important for people with complex health and social challenges, such as people with concurrent substance abuse and mental health problems, henceforth called “service recipients” ( Mueser *et al.* , 2003 ). They often have complex and multiple needs in connection with their substance abuse and mental health challenges, in addition to physical health problems ( Dickey *et al.* , 2002 ) and social challenges related to housing, finances and employment.

Internationally, the NPM doctrines have shaped the structures and practices of municipal health and social care organizations, but there are few empirical studies of the actual effects of NPM reforms on public service provision ( Lapuente and Van de Walle, 2020 ). At the same time, there is a growing recognition that the organizational and financial structures are impeding patient access and outcomes ( Miller, 2016 ; Stokes *et al.* , 2018 ). The aim of this article is twofold. The first part is empirical, where we investigate how organizational and financial structures and professional practices hinder and enable service integration. The second aim is theoretical, where we aim to explore public management values' influence on structures and practices in relation to service integration. The question to be answered is as follows: how do public management values influence service integration in municipal health and social care organizations?

Norway follows a welfare model where public financing and high and equitable service quality standards are key factors ( Kautto *et al.* , 2001 ). Through the well-established principle of local self-government ( Baldersheim and Ståhlberg, 2002 ), the ambitions of the Norwegian welfare state are to a great extent carried out in municipalities that vary in organization, size, finances, geography, resources and infrastructure ( Baldersheim and Ståhlberg, 2002 ). While Norwegian municipalities in principle have considerable responsibility for public health and social care, they are dependent on state allocation and reallocation to provide services ( Sørensen *et al.* , 2007 ).

### Theoretical framework

Health and social care reforms carry values that change organizational and financial structures, and these structures provide the division of tasks and hierarchies that determine professional practice and discretion ( Drucker, 2007 ). This means that the structures, by effectively limiting professionals' power, control their ability to make choices among possible courses of action. In this way, the structures can both constrain and enable professionals in assessing, allocating and delivering integrated services.

There seems to be a lack of knowledge in terms of a suitable theory to link the concepts of public management values, organizational and financial structures and professional practices. Hence, our theoretical aim seeks to tie these concepts together and demonstrate the implications for service integration. Hood (1991) described core values that influence reforms and shape the organizational design of public services. According to him, values related to economy and frugality are often associated with NPM, with matching of resources to defined tasks and goals, and breaking up organizations into single- or few-purpose units. Such values emphasize trimming of expenditure, frugality of resource use and heavy emphasis on economic reporting, and they are often carried out in organizations with mechanistic structures ( Hood, 1991 ). They also have a reputation for having a negative effect on other important values. On the other hand, values associated with the post-NPM goal of integration are flexibility, adaptivity, robustness and resilience. Capacity for resilience is related to the extent to which interdependent parts of the system are integrated in decision and information terms ( Hood and Dixon, 2015 ). A suitable organizational design for such values is one that recognizes that a certain degree of slack is needed in order to provide spare capacity in uncertain environments ( Vabø, 2009 ) and with a task division structure organized for collective thinking rather than silo thinking ( Hood, 1991 ).

Hood's theory on public management values focused on the shaping of organizations' structural design. We propose that service integration needs to be conceptualized in terms of structures *and* professional practices driven by values that promote integration. We aim to elaborate on Hood's theoretical concept of core values by showing how these values shape and affect both structures and professional practices, and why this has implications for *service* integration. We do this by applying the public management values associated with reforms labeled NPM and those labeled post-NPM to the findings. These are henceforth called frugal and economic values and integrative values. In the discussion, our findings inform how different combinations of frugal and economic values and integrative values within organizational and financial structures and professional practice either conflict or harmonize and therefore have different implications for service integration. As public management values are drivers for change in the field of service delivery ( Ackroyd *et al.* , 2007 ), the study of these can enhance understanding of integration in the complex and unstable environment in which public health and social care services operate ( Jørgensen and Bozeman, 2007 ).

## Research design and methodology

As we wanted to dig deep into service integration processes and bring evidence together from multiple sources, qualitative case study was a suitable design. Moreover, our aim to use the in-depth understanding of a “case” to build theory and offer new contributions to the existing knowledge base was coherent with the aims of case studies ( Yin, 2009 ). Public management values' influence on service integration in municipal health and social care organizations formed the case, with three Norwegian municipalities as case organizations. In order to gain sufficient amount of information about integration processes, we used purposive sampling to include municipalities with diversity in organizational structure and professional practice ( Seawright and Gerring, 2008 ). These two elements were given equal status throughout the research process and enabled understanding of the researched phenomenon. In this study, the degree of complexity in organizational and financial structures and professional practices increased with the size of the municipality.

### Context and participants

Municipality 1. Approximately 3,000 inhabitants and 15 people registered with concurrent substance abuse and mental health challenges. Mental health and substance abuse services are organized, financed and managed as one department under a larger unit with a unit manager. The municipality has no specific housing for the target group. Physical healthcare is provided by home nursing and one care facility.

Municipality 2. A population of approximately 30 000 and around 100 people registered in the target group. There is a unit for mental health and substance abuse, led by a unit manager. The services are divided into a mental health department, a substance abuse department and a staffed housing complex for people with mental health diagnoses; all of these have individual department managers and budgets. There is also an assessment team, which collaborates with specialist substance abuse services. Physical healthcare is provided by home nursing and several care facilities.

Municipality 3. Approximately 52 000 inhabitants and over 300 people registered in the target group. The organizational structure is complex. One unit contains all the mental health services: a mental health department, a staffed housing complex, three activity centers, one Flexible Assertive Community Treatment (FACT) team and one mental health and substance abuse outreach team. The departments and teams have separate budgets, but the FACT team has joint funding with the district psychiatric center, which provides specialist healthcare. Here, substance abuse services are organized as a department under the Norwegian Labor and Welfare Administration. Physical healthcare is provided by home nursing and various care facilities.

The empirical data consist of observations of 12 meetings (see Table 1 ) and 16 semi-structured in-depth interviews with 17 professionals, department managers and unit managers (see Table 2 ). Recruitment was purposive, through the managers of these services ( Creswell and Poth, 2018 ). Data collection took place from May to August 2019.

When seeking meetings to observe, we wanted to include interprofessional and interagency meetings where services for people with concurrent substance abuse and mental health challenges were discussed, assessed and allocated and where decisions were made. Managers who knew the organizations well were responsible for selecting the meetings, and the size of the municipality was a contributing factor in the selections. In the small Municipality 1 , there were few interprofessional and interagency meetings to observe. This resulted in opportunities to observe two meetings where service recipients also participated, although this was not a selection criterion. The same opportunity did not occur in Municipalities 2 and 3 . The observations were performed by the first author, with an overt nonparticipant observation role ( O'Reilly, 2012 ). Field notes were taken and coded to maintain participant anonymity. In our presentation of observational data, the service recipients have been given pseudonyms.

For the interviews, we recruited participants with over two years of experience and substantial knowledge of the services, whose work involved assessment and allocation or management. The recruitment resulted in seven professionals, seven department managers and three unit managers, all of whom had influential power in the observed meetings. We conducted the interviews at the participants' workplaces. The interview guide contained three themes concerning organizational and financial structures and professional practices, with follow-up questions depending on what the participants stated. All interviews were individual, except for one where two consultants wished to be interviewed together, due to their work schedule. Interviews were audiotaped, transcribed and anonymized.

The study was approved by the Norwegian Center for Research Data (ref. no. 300488), and exemptions from the duty of confidentiality were granted by the South-Eastern Norway Regional Committee for Medical and Health Research Ethics (ref. no. 2019/299 REK Sør-Øst). Comprehensive information about the study was provided orally and in writing to managers in the departments and units of interest and distributed to the participants. The participants signed an informed written consent.

### Analysis

**Figure 1:**
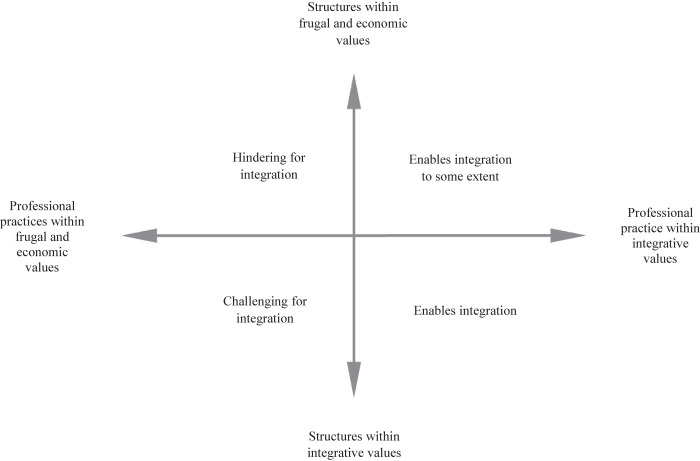
Table for deductive analysis

The dataset, consisting of observation reports and transcribed interviews, was analyzed both inductively and deductively. There was a dynamic interplay between the observation reports and the interviews, where the latter informed the processes observed in the meetings. This gave us an opportunity to find descriptions and explanations for the observed events in the interviews, and of that reason, both types of data were analyzed synchronously. In the inductively driven coding process for the empirical part of our aim, we were inspired by a stepwise deductive-inductive approach ( Tjora, 2019 ). In this phase, we searched the material for patterns and discussed the development of codes, code groups, themes and concepts. The initial analysis resulted in 244 empirically close codes, which we developed into five code groups. For example, the empirically close codes “The municipality has no staffed housing for people with substance abuse problems” and “We lack sufficient resources in terms of housing and staff” were categorized in the code group “Financial aspects affecting integrated services in housing and care facilities”. Later, the code groups were recategorized and refined, resulting in three main themes: (1) variations in approaching and organizing integration in mental health and substance abuse services, (2) structures and practices hindering access to physical healthcare and (3) structures and practices hindering allocation of housing. The findings in these main themes are presented by each case organization in the results. As public management values influence structures and practices ( Drucker, 2007 ), we were interested in studying how they specifically influence integration of municipal health and social care services. To explore this in the deductive phase, we applied public management values ( Hood, 1991 ; Hood and Dixon, 2015 ) to the findings. To work systematically, we made a table (see Figure 1 ) that shows four different combinations of public management values in organizational and financial structures and professional practices and searched the material for representations of these combinations. By doing this, we could address the possibilities and limitations the values have for service integration and thereby outline some implications.

## Findings

### Variations in approaching and organizing integration in mental health and substance abuse services

The small size of Municipality 1 enabled professionals to maintain a good overview of the service recipients' situation and of their service providers. In the integrated mental health and substance abuse department, we observed that the professionals utilized their respective competencies through daily discussions in order to help and guide each other and performed interprofessional assessments of new service applicants.

I think we've now found a system that works. It's all based on consent. That puts the patient in the center. They're the ones who control this. (Department manager, M1)

Putting the service recipient in the center laid the groundwork for service user involvement. The following extract demonstrates this approach:

Together in a meeting with Bobby were his municipal substance abuse counselor and a social worker from a specialist outpatient clinic. They were planning for detoxification and rehabilitation. Bobby talked about the changes that had happened after he started receiving municipal help. He had gotten a better and tidier apartment, was eating healthier food and had sorted out some of his finances, and now he felt more motivated to become drug-free. The substance abuse counselor kept Bobby's needs, worries and motivations at the center of their discussion by asking questions, supporting him and adjusting the plan according to his needs. (Extract from field notes)

The person-centered approach was noticeable through the involvement of Bobby and constant adjustments throughout the meeting. When asked about how the substance abuse counselor approached Bobby's mental health challenges, she explained as follows:

I ask questions to my co-workers in department meetings, since I do not have further education in mental health. We help and guide each other in our respective cases. (Professional A1)

The structural division in Municipality 2 into two mono-professional departments meant that professionals and managers could more easily help people with well-defined and relatively simple needs than those with complex needs. The department managers alone decided which department new service applicants were to belong to, based on what they perceived to be the dominant problem. It was considered too costly to provide services from both departments simultaneously.

We have to think about what the patient needs. And then money's part of it, because they're not really supposed to get both mental health and substance abuse services at the same time. (Professional A11, substance abuse department)

Although we observed in meetings that the service recipients often had dual challenges, they received services for either their mental health needs or their substance abuse.

The departments were organized as one unit; *however, they* never integrated resources.

There's no reason why I cannot ask someone in the substance abuse department to work in the mental health department. But we do not have a lot of resources, and I'll only move someone if they want to move. (Unit manager M6)

The integrative possibilities in the structures were not utilized. The co-location allowed for collaboration between the departments, an opportunity that was only used in ad hoc situations and not through systematic information exchange and decision-making.

Municipality 3 had many service recipients with complex life situations, a variety of services to offer and different ways to access them. This meant that people often received services in an arbitrary manner. The substance abuse department rarely found that people applied for their services. It was rather a situation where people approached them if they had financial or housing problems. By contrast, the mental health department received many applications, which were assessed comprehensively.

They describe their wishes and needs to the municipal services, and then we have to find out from where, from the lowest to the highest level, we can cover it. (Professional A9, mental health department)

Professionals and managers arranged various interagency meetings in order to coordinate services, exchange information and make decisions across the structural divisions. Despite the meetings, coordination and continuity was challenging. A professional in an observed meeting expressed as follows:

We have to reflect upon the fact that these people are very sick. We have to see the consequences of actions and decisions, and approach the entirety of the situations. (Extract from field notes)

The professional experienced that the services failed to provide continuity for severely ill service recipients. Meanwhile, another integrative mean was the establishment of interprofessional teams. One manager characterized the framework for interprofessional outreach teams as advantageous for service recipients.

They're like glue and lightning conductors for many other services. They do not have many clients compared to other services and they work evenings too, so I reckon the clients who have that team are lucky *.* (Manager M11, mental health department).

The framework enabled teams to be flexible in the time they spent on service recipients, which they argued was necessary to adapt to the complexity and uncertainty in service recipients' lives. This involved taking responsibility for coordination and being supportive and actively involved in contact with other relevant services. Because of this, the professionals in the outreach team prioritized building close relationships with their partners within and outside municipal services.

### Structures and practices hindering access to physical healthcare

Professionals in all three municipalities reported challenges when service recipients also had physical healthcare needs. The criteria for access as well as attitudes towards the service recipients could make access difficult.

As many of the mental health and substance abuse staff only worked day shifts, responsibility for service recipients' unanticipated needs was often handed to home nursing, which had round-the-clock services. Home care nurses reported challenges in fulfilling these responsibilities.

The necessary information is unavailable. We work 24/7 and we have to deal with people in all kinds of settings. We feel insecure if there is not good enough information about how they function and about the challenges. (Manager M4, home nursing)

The lack of available information could result in professionals feeling insecure with service recipients, which could affect the quality of service provision.

Municipality 1 had one care facility with good capacity for new patients and was not as strict regarding admission criteria as the two other municipalities. However, the professionals reported that the facility lacked competent staff to take care of service recipients with severe mental health or substance abuse problems. Since the municipality lacked a suitable care facility for these service recipients, they had to pay for admission to private facilities.

Even though they had a variety of care facilities and residential care homes, some of the participants in Municipality 2 described barriers to access for service recipients.

There are no residential care homes for people with mental health problems or substance abuse and they are not a group that necessarily fits in or is wanted everywhere. (Manager M5, substance abuse department)

This quote points to certain criteria for fitting in and that these people may be unwanted, which are barriers to access to general healthcare facilities. One manager described the decision-making processes for such facilities as a struggle. Due to both criteria and attitudes, service recipients were thrown back and forth in the system, experiencing multiple rejections.

A discussion concerning Peter's situation in an interagency meeting based on statements of concern showed similar processes in Municipality 3 :

Present were managers of five organizational units. Peter was 67 years old and lived in a private for-profit staffed housing complex due to his mental illness. He did not fulfill the criteria for continuing to live there, as he had developed a need for physical healthcare, which the mental health professionals did not provide. Peter was on a waiting list for admission to a care facility for people with substance abuse, even though he was currently clean. One manager questioned this, as it meant that he did not fulfill the admission criteria there either. Another manager expressed frustration that he “fell between two stools.” Eventually, they decided on a short-term stay in a general care facility, although they were aware of the unfortunate consequences for Peter, due to staff numbers and lack of competence. In the end, a manager suggested that it would be better if Peter was in active substance abuse, as this would make him entitled to the admission he had applied for initially. (Extract from field notes)

This can be understood as an example of adapting service recipients to organizational structures, instead of structuring the services according to service recipients' needs. In Peters' case, his needs always diverged from the criteria.

### Structures and practices hindering allocation of housing

All three municipalities experienced an increase in the number of service recipients, due to different reforms and incentives. One example was the process of downscaling residential institutions. This reform shifted the responsibility for severely ill people from specialist healthcare to municipal services and increased pressure to allocate staffed and independent housing. In addition to fulfilling a common basic need, people's homes were also a setting to receive municipal care services. For people with complex situations, who were not entitled to treatment in specialist healthcare, municipalities occasionally paid for staffed housing in private facilities.

The main challenge for Municipality 1 was its status as small and rural. There were no homeless people, but also no municipal staffed housing. The mental health and substance abuse department was responsible for two people for whom they had to pay for housing in private facilities.

Because the person was declared untreatable, specialist healthcare could not pay for it. *It* soon broke our budget, because we do not have that money. (Unit manager, M2)

When this person was declared untreatable, the responsibility was shifted to the municipality. The money for this was to be found within the unit, which led to overspending. Accordingly, this situation required cost cutting in other departments and an extra focus on prioritizing resources. As the municipality was very small, the establishment of staffed housing was not an option.

Municipality 2 had staffed housing for people with mental illness, but none for people with substance abuse problems and had no financial resources to pay for housing in private facilities. Instead, the professionals tried to meet the service recipients' needs with home-based services, as this was considered more cost-effective. Not all service recipients could handle such a solution, as they were too unstable to be able to live alone and receive help from different providers simultaneously. Managers close to service delivery level who were responsible for the budget felt pressure in such situations.

There's a lot of silo thinking here in decision making. And they really make you responsible for your budget. I often find people shift responsibility for particular service recipients. Especially when they cost a lot of money. (Manager M8, physical healthcare)

The complex situations of some service recipients necessitated comprehensive services, generating high costs. Because a well-balanced budget was a key measure of success, the managers and professionals tried to negotiate themselves out of taking responsibility for service recipients, thus protecting their finances. The following quote illustrates this protective behavior:

In meetings, it's easy to pass the buck. You do not say, ‘How can we solve this together?’ I mean the holistic approach. A bit more willingness and effort to help the person to get better. And then we have to work together. And I think that's all connected to the system. (Professional A12, mental health department)

This professional observed that shifts of responsibility were common in decision-making processes and called for both attitudinal and structural changes.

In Municipality 3 , we observed John's situation being discussed in a housing meeting. This illustrated the demanding situation and the possible unintended consequences of a lack of independent and staffed housing for service recipients.

John had a heavy substance abuse problem and a severe psychotic disorder. He had recently been discharged from hospital and was on a waiting list for admission to private nonprofit staffed housing. A manager who knew John well expressed emotional and engaged thoughts about many service recipients being too sick to endure long periods of waiting for housing. John had been discharged against the manager's will. The manager had been furious in a meeting with the hospital earlier that day, advocating for John's need and right to treatment. Due to the lack of suitable municipal apartments during the waiting time for admission, John was left homeless and became more psychotic. Now, the manager sadly stated, John had been missing for quite some time, and the manager was afraid he was dead. (Extract from field notes)

In this case, the manager expressed his concerns about how the lack of housing could lead to tragic outcomes. He wished for a greater emphasis on holistic and integrated approaches *to* prevent similar situations in the future.

Due to the lack of staffed housing, the municipality purchased such services in private facilities. The units and departments never integrated resources, and the process of deciding which unit or department should pay for these costly services was often left to the managers close to direct service delivery:

It's not good when the budget for substance abuse is here and dual disorders are both mental health and substance abuse. Sometimes we discuss what actually came first so we can avoid paying. (Manager M9, substance abuse services)

The discussions referred to in the quote were a common way of trying to avoid financial responsibility for services provided to certain service recipients. The decision-making processes were described as shifts of responsibility in an attempt to protect the budget of each unit. This was regarded as ethical dilemmas, which often hindered service recipients' access to suitable housing.

## Discussion

The findings draw a picture of professionals being unable to fulfill their mandate on behalf of the service recipients. Opportunities to assess, allocate and deliver integrated services were limited because the most important aim was to meet the financial goals and avoid red numbers in the budgets. At the same time, we found that when organizational and financial constraints were reduced and the context was small and manageable, as in *Municipality 1* *,* it was easier for professionals to meet service recipients' needs with person-centered and integrated service approaches. In contrast, the two larger municipalities with greater complexity in organizational and financial structures had stronger hierarchical management and barriers to integration and needed to be more frugal in decision-making processes. However, the largest Municipality 3 had a greater range of means at its disposal to enable integration, such as coordination through interagency meetings and establishment of interprofessional teams with strong coordination functions. Finally, we found that there were challenges for Municipality 2 , who was in the middle ground between simple and complex organizational structure. It was too large and complex to possess the same contextual advantages for integration as Municipality 1 and too small and simple to have such a wide range of integrative measures as Municipality 3 .

We found hindering and enabling factors for service integration in both organizational and financial structures and professional practices and were interested in studying how they are influenced by public management values. Our theoretical aim is to link the concepts of public management values, organizational and financial structures and professional practices and demonstrate the implications for service integration. We found four different combinations of frugal and economic values and integrative values in structures and practices, which we will further elaborate on below.

### Frugal and economic values in both structures and professional practices

When organizational structures are characterized by frugal and economic values, these strongly articulated values incentivize the professionals or force them to adapt to these structures with the same values. This combination of values results in territorial behavior, preventing professionals, units and departments from sharing and integrating resources ( Axelsson and Axelsson, 2009 ).

For home nursing in the three municipalities, accessible and available information is part of the organizational support structures essential for feeling safe in interaction with service recipients. Disaggregation, an outcome of organizational structures within frugal and economic values, challenge sufficient information exchange, as units and departments are organized with different purposes, budgets and management, creating single-purpose units ( Ashkenas *et al.* , 2002 ). Such structures reduce the capacity for resilience, as the professionals act independently of each other. When home care nurses lack helpful organizational and financial structures and an organizational culture with values that promote collaboration, integration of home care will be hindered.

The value of frugality by matching resources to tasks and goals materializes as criteria for access to physical healthcare facilities in Municipality 2 and 3 . In this way, some service recipients, such as Peter, neither fit in nor are welcome. Due to separate financial and management structures, the professionals lack common objectives, which creates incentives to shift service recipients and costs to other units and departments ( Kalseth *et al.* , 2015 ) rather than encouraging interdependency in decision making. This often results in rejections of service recipients, which can be regarded as structural stigmatization ( Knaak *et al.* , 2017 ), an unfortunate outcome due to frugal values within the structures. The financial structures in particular are too tight around the discretionary latitude. This prevents professionals from adequately assessing needs for integrated services ( Ponnert and Svensson, 2016 ) because the frugal values in the structures simply will not allow it.

Similar processes also occur regarding allocation of staffed housing in the same two municipalities. Since they do not have municipal staffed housing facilities, they consider buying this from private for-profit organizations if the service recipient has a severe and complex life situation. This is a solution that generates high costs, and the professionals are forced to adapt to the organizational and financial situation with frugal strategies by trying to meet service recipients' needs with comprehensive home-based services instead. Although the latter solution is considered the most cost-effective, both solutions generate high costs, which encourages territorial behavior and shifts of responsibility ( Axelsson and Axelsson, 2009 ), instead of preparing for interdependency between professionals. This behavior usually entails discussing whether the mental health challenges or substance abuse challenges are most dominant in an attempt to avoid responsibility for the financial burden involved. This, in turn, has proven to have a major impact on which services people receive and which they are unable to access ( Groenkjaer *et al.* , 2017 ).

Despite displaying frugal values in decision-making, professionals and managers call for integrated approaches, as in the tragic case of John. This demonstrates the conflict between values within organizational and financial structures on the one hand and values within professional practices on the other hand. Frugal values challenge professionals' opportunities to exercise discretion in order to work in an integrated manner because their discretionary latitude is limited. The financial structures in particular ensure that the decision-making processes are consistent with the objective of cost-efficiency and balancing budgets, which implies that professionals have few alternative courses of action to frugal strategies.

### Frugal values in structures and integrative values in professional practices

In this combination of values, we find frugal and economic values within the organizational and financial structures and integrative values in the professional practice. The managers and professionals adapt to the frugal organizational and financial structures by establishing integrative initiatives, which may enable integration in some cases, as found in Municipality 3 . This large municipality, consisting of many independently structured units and departments, with sharply defined responsibilities, forms a highly complex and fragmented organizational landscape ( Glouberman and Mintzberg, 2001 ). The complexity makes integration difficult when professionals attempt this through coordination in interagency meetings. At the same time, the coordinating role of professionals in interprofessional teams is an important contribution we bring to the research on integration. These teams have organizational structures where health educated and social care educated staff are integrated, and they have more robust financial structures than in the rest of the service system. In this way, the municipality creates smaller organizational units where professionals work interdependently, within an otherwise complex and fragmented organizational landscape. When coordinators work within such structures, they are able to be flexible and adaptive ( Vabø, 2009 ) and to breach the structural boundaries for the service recipients ( Santos and Eisenhardt, 2005 ), all of which are important values in integrative work. This is done by prioritizing close professional collaboration, as the coordinator builds integrated networks in processes with and around service recipients ( Darcis and Thunus, 2020 ). This gives us reason to argue that when the values robustness, flexibility and adaptivity are emphasized in professional practice, service integration can be enabled.

### Integrative values in structures and frugal values in professional practices

In this combination, we find the integration, robustness and flexibility in organizational structures, which accommodate integration. Professionals and managers adapt to these structures with frugal and economic values, associated with NPM. Findings in this relationship can indicate that although the structures accommodate integration, professionals and managers think and act based on the values of NPM doctrines ( Lapuente and Van de Walle, 2020 ).

The mental health and substance abuse services in Municipality 2 are co-located and organizationally structured as an integrated unit with a unit manager. Although integration of financial resources has proven to increase robustness and promote collaboration ( Tsiachristas *et al.* , 2013 *)* , the services in Municipality 2 are managed and financially structured as two mono-professional departments *.* In the absence of management that promotes visions and goals for the entire unit, the professionals continue to work in silo fashion, even though they are co-located ( Bussu and Marshall, 2020 ). Their poor collaboration in information exchange and decision-making and the structures of financial distribution constrain their capacity to achieve integration. In the case of Municipality 2 , this results in service recipients receiving services for either challenges related to substance abuse or mental health. When services is marked by an authoritarian barrier to collaborative culture in the hierarchical structures ( Hood, 1991 ), the result is a professionally fragmented service system with no professional interdependency, which is highly detrimental to service integration ( Willumsen *et al.* , 2012 ).

### Integrative values in both structures and professional practices

When the values represented by organizational and financial structures and professional practices are both integrative values, integration is enabled. We find this combination of values in Municipality 1 , who due to the small and easily overviewed context have a relatively simple organizational structure with resources and staff for both mental health and substance abuse services integrated in the same department. This prepares for interdependency, which allows professionals to exercise discretion together and take alternative courses of action, instead of exercising territorial behavior. As financial constraints have proven to counter adaptivity ( Vabø, 2009 ), we demonstrate that financial robustness through integrated resources enables professional practice to be flexible and adaptive. This implies that removing the financial barriers between mental health and substance abuse services can enable integration, which in turn allows for a person-centered service approach, as in the meeting with Bobby.

The robustness is also recognized in care facilities, where good capacity minimizes the need for strict criteria-based access, except for service recipients with severe and complex problems, for whom it is necessary to pay for private facilities. Further, with integrated organizational and financial structures between mental health and substance abuse services, shifts of responsibility become a nonissue. This implies that integrative values in the structures reduces the tensions that often prevent professionals and managers from co-creating values essential for meeting service recipients' needs ( Anderson *et al.* , 2016 ), while also increasing their capacity for resilience.

The small context of Municipality 1 entailed methodological limitations for this study. The small population meant that we could observe two meetings involving service recipients. As we had no other options for observations, we had to use the few opportunities provided. In the other two municipalities, we did not observe meetings with service recipients present, which may be seen as detracting the credibility for this study. However, in addition to being a methodological explanation for the selection, it can underscore the fact that size is actually an important factor. At the same time, it could very well be that the small, transparent context, along with the effects of the integrative values in both structures and practices, allows professionals to take alternative courses of action, including in research collaborations such as this. Future studies are needed in order to provide more substantial answers. Another limitation is that the findings do not enable generalization to all municipalities that fit the descriptions of the municipalities in this study. We are, however, able to make an analytical generalization based on organizational and financial structures and on how they control professionals' ability to exercise discretion. Our study provides theoretical and practical insights into the implications of public management values for service integration but might also provide impetus for further research into this complex research area.

## Conclusion

Integrated services are aimed to be accessible for individuals with complex and comprehensive needs, yet this is a challenge for most welfare states. Although our empirical field is Norwegian municipal health and social services, this problematic area may be of interest for other countries. Our theoretical contribution indicates that public management values have implications for organizational and financial structures and professional practice. Here, we argue that integration is impeded by the frugal and economic values in NPM doctrines that still strongly influence organizational and financial structures as well as professional practices in Norwegian municipal health and social care services. Regarding the implications for policymakers and managers, our contribution points out the incompatibility between the demands of economic management principles and the goal of integration. Professionals are bound and tied by the rope of frugality, giving them few alternative actions to frugal strategies. At the same time, we find examples of how the post-NPM goal of integration is realized when either or both of the organizational/financial structures and the professional practices are fused with integrative values. In order to enable integration, the ropes of frugal and economic values must be loosened up, allowing professionals to use their discretional abilities to assess, allocate and deliver integrated services. This implies that the values of flexibility, adaptivity, robustness and resilience must be at the forefront of organizational and financial structures and professional practices. Additionally, interdependency between organizational units and professionals is important for service integration. This seems to be more achievable within small and relatively simple organizational structures as opposed to large and complex organizational structures.

## Figures and Tables

**Table 1:** Observed meetings

Municipality	Title of meeting	Number of participants	Service recipient included	Interprofessional	Interagency
1	Responsibility team meeting	3	Yes	Yes	Yes
1	Responsibility team meeting	3	Yes	Yes	Yes
1	Decision meeting	5	No	Yes	No
2	Department meeting, mental health	4	No	No	No
2	Department meeting, addictions	5	No	No	No
2	Decision meeting, mental health	2	No	No	No
2	Decision meeting, addictions	2	No	No	No
3	Meeting based on statements of concern	5	No	Yes	Yes
3	Housing meeting	6	No	Yes	Yes
3	Housing meeting	8	No	Yes	Yes
3	FACT meeting	7	No	Yes	No
3	Collaborative meeting concerning patients being discharged from hospital	13	No	Yes	Yes

**Table 2:** Participants for interviews

Municipality	Educational background	Role	Department/unit	Age	Sex
1	Nurse	Professional	Mental health and substance abuse department	50–59	F
1	Nurse	Department manager	Home nursing	50–59	F
1	Nurse	Unit manager	Unit for family and health	60–69	F
1	Social educator	Department manager	Mental health and substance abuse department	50–59	F
2	Nurse	Professional	Department of mental health	40–49	M
2	Social worker	Professional	Department of substance abuse, and assessment team	50–59	F
2	Nurse	Department manager	Department of mental health	40–49	F
2	Nurse	Department manager	Department of substance abuse	40–49	F
2	Nurse	Unit manager	Unit for substance abuse and mental health	60–69	F
2	Social educator	Unit manager	Unit for generic services	50–59	F
3	Nurse assistant	Professional	Mental health and substance abuse team	50–59	M
3	Social educator	Professional	FACT team	40–49	M
3	Nurse	Professional	Department of mental health	30–39	F
3	Nurse	Professional	Department of mental health	40–49	F
3	Social educator	Department manager	Department of mental health	30–39	M
3	Nurse	Department manager	Department of substance abuse	30–39	M
3	Nurse	Department manager	Home nursing	50–59	F
